# 1901. High Prevalence of Latent *Tuberculosis* Infection in a Malaysian Prison

**DOI:** 10.1093/ofid/ofad500.1729

**Published:** 2023-11-27

**Authors:** Lu Zhang, Sheela Shenoi, Adeeba Kamarulzaman, Sangeeth Dhaliwal, Ahsan Ahmad, Frederick Altice

**Affiliations:** Yale University, Connecticut; Yale University, Connecticut; Yale University School of Medicine, Kajang, Selangor, Malaysia; Yale University School of Medicine, Kajang, Selangor, Malaysia; Yale University School of Medicine, Kajang, Selangor, Malaysia; Yale University School of Medicine, Kajang, Selangor, Malaysia

## Abstract

**Background:**

Given the rising tuberculosis (TB) incidence and mortality, strengthening the identification and treatment of those with latent tuberculosis infection (LTBI) is urgently needed. Prisons are a TB hotspot but there is a paucity of data on LTBI in prisons. This study characterized LTBI in the largest prison in Malaysia.

**Methods:**

From 22^nd^ October 2019 to 19^th^ January 2023, adults newly admitted (< 2 days) to the Kajang men’s prison without current active TB (excluded by negative culture, Acid-Fast Bacillus smear, or GeneXpert results) or a history of active TB in the past 2 years, were invited to participate. Enrollees underwent tuberculin skin tests (TST), chest X-ray, and blood tests for HIV, hepatitis C (HCV), hepatitis B, and c-reactive protein (CRP). Sociodemographic variables and drug use history were collected by questionnaires. LTBI was defined as TST size ≥ 5mm for people living with HIV and ≥ 10mm for people living without HIV, without evidence of active TB. Multivariable logistic regression was performed to identify independent correlates of LTBI by stepwise backward regression with inclusive factors from univariate analysis (p< 0.1). Multicollinearity was checked by the variance inflation factor (VIF).

**Results:**

We enrolled 601 eligible people deprived of liberty, with a median age of 42 (IQR 36-50), 8.8% living with HIV, 43.4% with HCV infection. The prevalence of LTBI was 68.2% (95%CI: 64.4%-71.8%). The independent risk factors for LTBI overall (Table 1) included opioid use disorder, history of homelessness, and HCV. Marijuana use was associated with decreased risk of LTBI among all cases and among people with HIV. For people without HIV, HCV and CRP≥ 5mg/ml were independent risk factors for LTBI, while methamphetamine use was associated with a reduced risk of LTBI.

**Figure:**
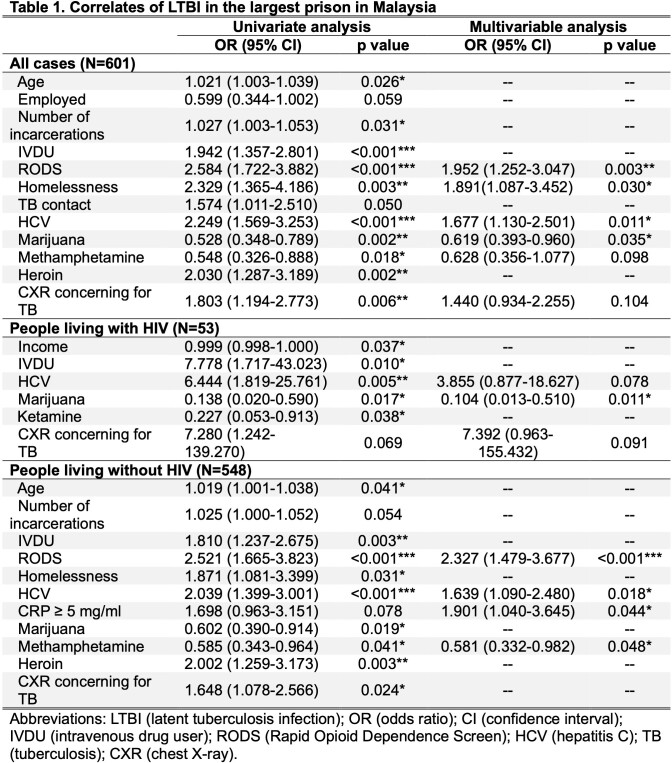

**Conclusion:**

The prevalence of LTBI in a Malaysian prison was high, necessitating the implementation of TB preventive therapy. The high prevalence of HCV may increase the risk of hepatotoxicity during LTBI treatment. Further data is needed on the risks of TB preventive therapy among people deprived of liberty.

**Disclosures:**

**All Authors**: No reported disclosures

